# Echocardiography in Autoimmune Rheumatic Diseases for Diagnosis and Prognosis of Cardiovascular Complications

**DOI:** 10.3390/medicina56090445

**Published:** 2020-09-01

**Authors:** George Makavos, Maria Varoudi, Konstantina Papangelopoulou, Eirini Kapniari, Panagiotis Plotas, Ignatios Ikonomidis, Evangelia Papadavid

**Affiliations:** 1 Second Cardiology Department, Attikon Hospital, Medical School, National and Kapodistrian University of Athens, 12462 Athens, Greece; mvaroudi@gmail.com (M.V.); kpapanel@gmail.com (K.P.); pplotas@upatras.gr (P.P.); ignoik@gmail.com (I.I.); 2 Second Department of Dermatology and Venereology, Attikon Hospital, Medical School, National and Kapodistrian University of Athens, 12462 Athens, Greece; kapniari_27@yahoo.gr (E.K.); papadavev@yahoo.gr (E.P.)

**Keywords:** autoimmune rheumatic diseases, echocardiography, three dimensional echocardiography, myocardial deformation

## Abstract

Autoimmune rheumatic diseases are systemic diseases frequently affecting the heart and vessels. The main cardiovascular complications are pericarditis, myocarditis, valvular disease, obstructive coronary artery disease and coronary microcirculatory dysfunction, cardiac failure and pulmonary hypertension. Echocardiography, including transthoracic two and three-dimensional echocardiography, Doppler imaging, myocardial deformation and transesophageal echo, is an established and widely available imaging technique for the identification of cardiovascular manifestations that are crucial for prognosis in rheumatic diseases. Echocardiography is also important for monitoring the impact of drug treatment on cardiac function, coronary microcirculatory function, valvular function and pulmonary artery pressures. In this article we summarize established and evolving knowledge on the role of echocardiography for diagnosis and prognosis of cardiovascular abnormalities in rheumatic diseases.

## 1. Introduction

Autoimmune rheumatic diseases (ARD) are immune-mediated diseases targeting the connective tissues. Cardiovascular complications are frequent and almost all cardiac structures may be affected. The main cardiovascular manifestations in ARD are (a) pericardial, myocardial and vascular inflammation, (b) coronary artery disease (CAD) and dysfunction of the coronary microcirculation, (c) structural and functional abnormalities of the heart valves and (d) elevated pulmonary artery pressures [1,2].

Anti-inflammatory treatment reduces inflammation and disease-associated morbidity and mortality. However, the incidence of cardiovascular (CV) events is higher in ARD patients in comparison with the general population resulting in worse prognosis [3]; thus, the early identification of cardiovascular abnormalities is crucial in order to improve outcomes.

Echocardiography is an established and widely available imaging technique for the detection of cardiovascular involvement and for monitoring the effects of treatment on cardiac and vascular structure and function in ARD patients. In this article, we aim to review the role of echocardiography for diagnosis and prognosis in ARD associated with increased incidence of cardiovascular complications and higher cardiovascular risk [4] including rheumatoid arthritis (RA) systemic lupus erythematosus (SLE), systemic sclerosis (SSc), psoriasis and psoriatic arthritis and ankylosing spondylitis (AS).

## 2. Cardiac Involvement in Autoimmune Rheumatic Disease

### 2.1. Rheumatoid Arthritis (RA)

Cardiovascular complications are common including (a) pericardial involvement (in 30–50% of RA patients) [5] but symptomatic in less than 1% [6], (b) myocardial involvement (in 3–30% of RA patients) [7] manifested as myocarditis (focal lymphocytic, diffuse necrotizing or granulomatous) and myocardial fibrosis leading to RA associated cardiomyopathy [6,8], (c) valvular heart disease (regurgitation in 21% and stenosis in 3%) [9] and (d) accelerated atherosclerosis and epicardial CAD. It has been demonstrated that positive auto-antibodies, high disease activity and the presence of conventional CV risk factors were all strongly associated with a high CV risk [6,10]. Angiographic evidence of CAD was present in 75% of RA patients by the age of 65 [1] and the risk of myocardial infarction was 2 to 3-fold higher than the risk in the general population [11,12,13]. Impaired coronary microcirculatory function and increased aortic stiffness may also be detected in the course of the disease [1].

### 2.2. Systemic Lupus Erythematosus (SLE)

Systemic Lupus Erythematosus is mostly complicated by (a) valvular heart disease (in 13–100%) [1,14], (b) pericarditis in 40% [15] and myocarditis (in 10%), possibly immune complex mediated with granular complement and immunoglobulin deposits [6,16] and (c) accelerated atherosclerosis, coronary microvascular dysfunction and coronary arteritis [17,18]. A 2 to 3-fold higher morbidity and mortality in SLE patients compared with the general population due to the aforementioned complications has been demonstrated [1].

### 2.3. Systemic Sclerosis

In Systemic Sclerosis (SSc) the main cardiovascular complications are (a) pulmonary artery hypertension (PAH) [19,20] and (b) myocardial involvement and fibrosis [21] either primary due to the dysfunction of the coronary microcirculation and primary systemic myositic disease or secondary due to PAH [22,23].

### 2.4. Psoriasis and Psoriatic Arthritis 

Psoriasis and psoriatic arthritis are characterized by (a) accelerated atherosclerosis and increased CV risk because of linked causative pathways with CAD including inflammation and oxidative stress [24] (in psoriatic arthritis, cardiovascular death is the leading cause of death (in 36.2% of patients) with a death risk 1.3 times greater compared with the general population [6]), (b) pericarditis (in 18.2%) [25], (c) myocarditis (in 15.9%) [25] and (d) subclinical myocardial dysfunction, impaired coronary microcirculatory function and increased aortic stiffness have also been reported in psoriasis patients to a similar degree with CAD patients [26].

### 2.5. Ankylosing Spondylitis (AS)

The main cardiovascular manifestations in AS include (a) aortitis with aortic and mitral valve involvement [25,27] and (b) increased risk of CAD associated with higher disease activity [4,6].

## 3. Echocardiographic Assessment of Cardiovascular Involvement in Autoimmune Rheumatic Diseases

The main echocardiographic findings consistent with cardiovascular involvement in autoimmune rheumatic diseases are summarized in Table 1.

## 4. Pericardial Involvement

In case of suspected pericarditis in ARD, the amount and location of pericardial fluid and the rare presence of tamponade and constrictive pericarditis can be assessed with two-dimensional echocardiography (2D echo) and Doppler imaging [28,29]. Swinging heart, early right ventricular diastolic collapse, late right atrial diastolic collapse, respiratory variation in ventricular chamber size, dilated inferior vena cava and exaggerated mitral inflow respiratory variability of >25% are findings consistent with cardiac tamponade [29]. Septal bounce exaggerated respiratory changes of the mitral E wave and Tissue Doppler mitral e’ velocity >8.0 cm/s [29] are findings indicative of constrictive pericarditis. However, the extent and location of pericardial thickening and calcification often cannot be reliably assessed with 2D echo. In such cases, cardiac CT can be used to detect pericardial thickening and the degree of the fibrocalcific process as well as the amount and location of pericardial fluid in challenging echo windows [29].

## 5. Myocardial Involvement

### 5.1. Rheumatoid Arthritis

As some studies have indicated that the systolic function of the LV may be moderately reduced in RA patients suggesting progression to heart failure [30], assessment of LV performance in relevance with the occurrence of symptoms of heart failure is very important. Global hypokinesis or segmental wall motion abnormalities can be detected with 2D echo, whereas assessment of diastolic function and LV filling pressures with Doppler echocardiography are also crucial [31,32]. In patients with normal ejection fraction (EF), a ratio of E/e’ >14, tissue Doppler e’ velocity of the interventricular septum <7 cm/s or tissue Doppler e’ velocity of the lateral wall >10 cm/s, left atrial volume index >34 mL/m^2^ and tricuspid regurgitation maximum velocity >2.8 m/s are findings consistent with elevated filling pressures [32]. Myocardial dysfunction has been associated with high disease activity and duration in RA [33,34].

Although extensively validated and used in daily practice, left ventricular ejection fraction (LVEF) is not the ideal marker for the evaluation of myocardial function because a reduction in LVEF is indicative of a relatively advanced myocardial dysfunction. Speckle tracking deformation analysis is considered a more sensitive technique for the identification of subclinical alterations in myocardial contractility, providing a more comprehensive study of myocardial function [35].

It has been demonstrated that subclinical myocardial dysfunction may be present in very early RA patients without CV risk factors. Impaired values of global longitudinal and circumferential strain were observed in RA patients with preserved ejection fraction and high disease activity compared with healthy controls [36,37].

Inflammatory cytokines including tumor necrosis-α (TNFα), interleukin-1 (IL-1) and interleukin-6 (IL-6) have a pivotal role in RA and may induce myocardial and vascular dysfunction and promote LV remodeling and fibrosis [38,39,40].

Speckle tracking deformation analysis can be applied in ARD patients with unexplained dyspnea and a preserved ejection fraction. Impaired global longitudinal strain (GLS) values in combination with echocardiography findings of elevated filling pressures in the absence of valvular disease or pulmonary abnormalities are consistent with heart failure with preserved ejection fraction [32,35]. Speckle tracking is also useful for the assessment of the effects of anti-inflammatory treatment in RA. It has been shown that RA patients compared with normal controls had impaired longitudinal (−18.5% vs. −22.50%, respectively), circumferential and radial strain [41]. Impaired longitudinal and circumferential strain values correlated with increasing values of inflammation markers (interleukin-1β) and markers of oxidative stress (protein carbonyl). In patients with RA and coexisting CAD, LVEF was lower compared with normal controls and RA patients without CAD. Lower LVEF values correlated with elevated values of oxidative stress markers (protein carbonyl, nitrotyrosine and malondialdehyde) [42].

**Figure 1:**
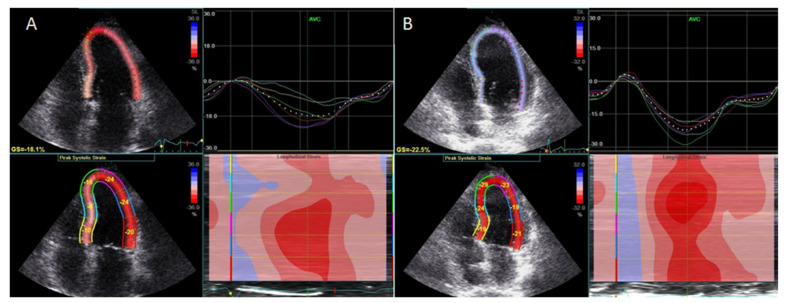
(**A**) A patient with rheumatoid arthritis and impaired global longitudinal strain (−18.1%). (**Β**) After treatment with the interleukin-1 inhibitor, global longitudinal strain improved (−22.5%).

Treatment with an interleukin-1 (IL-1) inhibitor (anakinra) resulted in improved LV global longitudinal strain (Figure 1).

LV twisting-untwisting in RA patients, in parallel with a reduction in oxidative stress markers and interleukin-6, particularly in patients with coexisting CAD [40,41,42,43], suggested improvement of myocardial function with targeted anti-inflammatory treatment. Finally, treatment with an IL-6 inhibitor (tocilizumab) resulted in a greater improvement of GLS and reduction in markers of inflammation C-reactive protein (CRP) and oxidative stress (protein carbonyl and malondialdehyde), compared with conventional synthetic disease-modifying antirheumatic drugs or glucocorticoids [44].

The impairment of circumferential systolic and diastolic strain has been associated with disease activity and the degree of inflammation and diffuse fibrosis as expressed by expansion of extracellular volume by cardiac magnetic resonance (CMR) T1 mapping [45,46].

Finally, LV hypertrophy is associated with an increased prevalence of cardiovascular events in the general population and in RA patients. TNFα seems to be a significant determinant of LV remodeling [47]. It has been demonstrated that treatment with the TNFα inhibitor etanercept may significantly reduce LV hypertrophy [48] and TNFα inhibition may improve longitudinal strain values in RA patients [37], suggesting the reversal of myocardial structural and functional abnormalities with anti-inflammatory treatments.

### 5.2. Systemic Lupus Erythematosus

Patients with SLE myocarditis may have global or segmental wall motion abnormalities by 2D echo and low systolic Tissue Doppler imaging velocities of the mitral annulus [6,49]. Impaired biventricular myocardial strain by speckle tracking has also been reported to be suggestive of subclinical myocardial dysfunction [50,51]. Impaired LV longitudinal strain values correlated with a more advanced degree of diffuse fibrosis by CMR T1 mapping [52].

### 5.3. Systemic Sclerosis

Transthoracic 2D echo is a valuable tool for the assessment of heart function in SSc, whereas 3D echo may offer additional information on ventricular volumes and EF especially in case of PAH affecting the right ventricle (RV). Structural and functional abnormalities of the LV and RV have been reported in up to 23% and 21% of SSc patients, respectively [53]. Primary SSc involvement of the myocardium typically affects the LV, leading to reduced LVEF (in 5.4%), LV hypertrophy (in 22.6%) and LV diastolic dysfunction (in 17.7%) [54,55]. Tissue-Doppler imaging and speckle tracking may detect subtle myocardial dysfunction in SSc [56,57].

Patients with SSc and preserved EF as compared with controls had impaired GLS (−18.2 ± 1.8% vs. −21.3 ± 1.7%, respectively (*p* > 0.01)) and circumferential strain values (−18.2 ± 2.3% vs. −21.3 ± 2.1%, respectively (*p* > 0.01)). In patients with SSc, impaired GLS and circumferential strain values each correlated with worse functional capacity and rhythm disturbances [57].

It has also been demonstrated that global longitudinal strain of the right ventricle is impaired in SSc patients compared with age and sex-matched controls (−17.7% vs. −20.4%, respectively) [58].

Impaired myocardial strain correlated with disease activity and the amount of focal and diffuse myocardial fibrosis by CMR T1 mapping [59] and has been associated with increased cardiovascular risk in SSc [60].

### 5.4. Psoriasis

It has been demonstrated that GLS is impaired in a similar degree in psoriasis and CAD patients compared with normal controls (−16.5% in psoriasis vs. −16.2% in CAD vs. −21.9% in controls). This is consistent with the presence of subclinical myocardial dysfunction in psoriasis due to a similarly elevated inflammatory and oxidative stress burden as indicated by elevated IL-6 and malondialdehyde values [26]. Treatment with an interleukin-12/23 inhibitor resulted in a greater improvement of GLS compared with treatment with a TNFα inhibitor or cyclosporine, in parallel with a reduction of IL-6, IL-12, IL-17, TNFα and malondialdehyde levels [61]. Moreover, the magnitude of GLS improvement correlated with the reduction in oxidative stress and inflammatory markers (IL-6, IL-12 and malondialdehyde). Treatment with interleukin-17 inhibitors also resulted in a greater improvement of systolic and diastolic longitudinal myocardial deformation markers and LV twist compared with cyclosporine or methotrexate treatment. Changes in myocardial deformation markers correlated with the degree of reduction of oxidative stress markers (malondialdehyde and protein carbonyl levels) [62].

## 6. Valvular Heart Disease

### 6.1. Rheumatoid Arthritis

Two and three dimensional transesophageal echocardiography is an accurate technique for the diagnosis and assessment of the severity of valvular heart disease [1,7,63]. Rheumatic valve nodules are detected in 32% of RA patients [7]. Valve nodules are oval shaped and small in size (4–12 mm) formations. Aortic and mitral valves are equally affected. Rheumatic nodules have homogenous echoreflectancy and regular borders and they are located at the leaflets’ basal or mid segments, the myocardium or chordae tendinae [7]. Moderate to severe valve regurgitation may be detected due to the thickening and prolapse of the mitral leaflets in the case of RA valvulitis [1]. Libman–Sacks-like vegetations are rarely observed [1].

### 6.2. Systemic Lupus Erythematosus

Valvular heart disease may be identified in 30–50% with transthoracic and 60–70% with transesophageal echocardiography in SLE patients [1]. Valvular disease is characterized by the thickening of the mid portions and the tips of the leaflets. Leaflet prolapse or perforation, chordal rupture and significant valvular regurgitation may be detected, whereas valve stenosis is rare [1]. 

Libman–Sacks vegetations are detected in 10% by transthoracic and 30–40% by transesophageal echocardiography in SLE patients. Libman–Sacks vegetations are typically located on the tips of the left heart valves as inflammatory formations of >2 mm in diameter, or thrombotic or firm masses that may resolve or relapse [1]. Thrombus formation on the vegetations may lead to macro or microembolic events [1]. Compared with mild valvular abnormalities, moderate to severe valvular dysfunction was associated with a 3–4 times higher rate of occurrence of symptoms of valvular disease, the need for valve surgery, stroke or other embolic events and death during a follow-up period of 2–8 years [1]. The prevalence of valvular abnormalities and other sources of embolism has been reported in up to 61% of patients with antiphospholipid syndrome with higher rates of thromboembolic events associated with higher anticardiolipin antibody titers of >40 GPL units [64]. Thus, patients with antiphospholipid syndrome and clinical symptoms of peripheral embolism or high anticardiolipin antibody titers should be examined with transoesophageal echo (TOE) [65].

### 6.3. Ankylosing Spondylitis

Aortitis is a frequent and serious complication of AS, occurring late in the course of the disease. The inflammatory process begins from the aortic root wall and may expand to the aortic cusps causing thickening and fibrosis [66]. Mitral valve and heart conduction systems may also be involved in the course of the disease [66]. The main echocardiography findings in the case of aortic involvement included aortic root thickening and dilatation and aortic cusp thickening [67], whereas aortic regurgitation has been reported in up to 34% of patients, mitral regurgitation in 1–76% and mitral valve prolapse in 5.7–10% [25,27].

## 7. Coronary Artery Disease

### 7.1. Rheumatoid Arthritis

RA is characterized by a higher incidence of CAD and cardiovascular events compared with the general population due to systemic inflammation and the high prevalence of traditional CV risk factors [11,68]. Patients with RA and extra-articular symptoms, rheumatoid factors-positive, extended disease duration, additional risk factors and the presence of carotid plaques by carotid ultrasonography are at increased CV risk; thus, they should be screened for CAD [69,70]. According to European League Against Rheumatism (EULAR) recommendations, the assessment of CV risk is recommended for all patients with RA and ARD at least once every five years and after major changes in antirheumatic therapy [69]. Non-invasive imaging techniques may have a key role for the evaluation of CV risk in ARD patients [70]. A multimarker approach including various imaging modalities has been proposed for the identification of ARD patients with subclinical atherosclerosis, permitting initiation of cardiovascular protective therapies (e.g., statins) to reduce CV risk [71,72]. 

The detection of carotid atherosclerosis has been shown to reclassify patients with RA into a higher risk group [73]. In ARD patients with symptoms of CAD, stress echocardiography may offer valuable information for the diagnosis and prognostic assessment of CAD [74]. It has a similar diagnostic accuracy compared with radionuclide stress tests; however, it lacks radiation exposure [74]. It has been indicated that patients with RA had a 2-fold higher rate of positive exercise echo for myocardial ischemia compared with controls and a positive stress echo was associated with increased disease duration. Moreover, 5-year all-cause mortality was 14.9% in RA patients with a positive stress echo compared with 4.3% in those with a negative stress echo for ischemia [75]. Additionally, silent myocardial ischemia in the absence of obstructive coronary lesions may be detected with stress echo in RA in a similar prevalence as in diabetes mellitus due to abnormal microcirculatory function [76]. 

**Figure 2:**
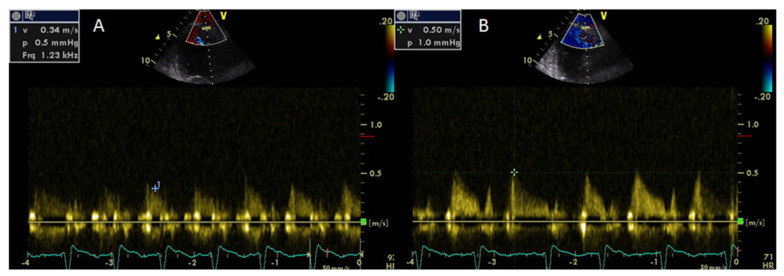
A patient with rheumatoid arthritis and impaired coronary flow reserve by Doppler echo. Coronary flow of the left anterior descending artery (LAD) at rest (**A**) and after adenosine infusion (**B**). Coronary flow reserve as a maximum diastolic velocity ratio was calculated at 1.5.

Coronary flow reserve can be calculated non-invasively by Doppler echocardiography of the left anterior descending artery as the ratio of peak diastolic velocity after adenosine infusion to peak diastolic velocity at rest [77]. Coronary flow reserve may provide information regarding the integrity of coronary microcirculation and the presence of obstructive coronary stenosis and is associated with a worse prognosis even in the absence of obstructive CAD [78]. A CFR value of >2 is consistent with significant epicardial coronary stenosis [79]. It has been demonstrated that the CFR is impaired in systemic rheumatic diseases compared with healthy controls [80]. Significantly lower CFR values have been found in untreated patients with early RA due to abnormalities of the coronary microcirculation [81] (Figure 2).

Decreasing CFR values correlated with elevated values of symmetric dimethylarginine, a marker of endothelial dysfunction that acts as an endogenous inhibitor of nitric oxide synthase [81]. 

Treatment with an interleukin-1 (IL-1) inhibitor (anakinra) resulted in improved CFR in RA patients, particularly when CAD coexisted, indicating a beneficial effect of the anti-inflammatory treatment on coronary microcirculatory function in RA [40,41,42,43].

### 7.2. Systemic Lupus Erythematosus

The calculation of CV risk based on the traditional risk scores is inaccurate in SLE [82]. Systemic inflammation and increased disease activity have a major role for the high incidence of atherosclerosis and CAD in SLE [17,83]. Atheromatic plaques were detected in 40% of SLE patients by carotid ultrasonography and plaque progression was greater in SLE patients compared with the general population [84]. Coronary flow reserve has been found to be impaired in young SLE patients without risk factors for CAD suggesting coronary microcirculatory dysfunction [85].

### 7.3. Psoriasis

Coronary flow reserve was similarly impaired in psoriasis and CAD patients after adjustment for atherosclerotic risk factors in parallel with elevated markers of inflammation and oxidative stress [26]. Moreover, treatment with IL-12/23 and IL-17 inhibitors resulted in a significant improvement of CFR, greater compared with TNFα inhibition, cyclosporine or methotrexate treatment. CFR improvement correlated with a concomitant reduction in the inflammatory and oxidative stress markers [61,62].

### 7.4. Ankylosing Spondylitis

Several studies have indicated an increased CV risk and accelerated atherosclerosis in AS [86,87]. A meta-analysis demonstrated a significantly increased risk for CAD in patients with AS with an 41% excess risk [88].

Inflammatory cytokines, oxidative stress, traditional CV risk factors and the toxic effects of non-steroid inflammatory drugs have been considered to cause aggravation of the atherosclerotic process in AS [89,90,91]. Echocardiography assessment of structural and functional abnormalities and especially stress echocardiography for detection of ischemia may provide significant information for risk stratification and clinical decision making in AS patients.

## 8. Pulmonary Hypertension

Transthoracic echocardiography is a first line modality for PAH screening and for the detection of RV dysfunction because RV remodeling has a major role in the prognosis of PAH patients [20]. Continuous wave Doppler measurement of TRVmax may reveal increased pulmonary artery systolic pressure. Moreover, indirect findings consistent with PAH such dilation and dysfunction of the right ventricle, systolic flattening of the interventricular septum, dilated inferior vena cava and increased pulmonary regurgitation early diastolic velocity may raise suspicion for the presence of PAH [20]. Exercise stress echo may identify patients at an increased risk to develop PAH. Patients with SSc, SLE or mixed connective tissue disease and a greater rise in mean pulmonary artery pressure during exercise had increased rates of PAH development in the future [92].

Moreover, poor contractile reserve and impaired GLS during exercise stress echo in SSc patients has been associated with reduced functional capacity and higher values of pulmonary artery systolic pressures [93].

Due to the complex triangular-crescent shape of the RV, a structural and functional assessment of the RV by 2D echo is based on geometrical assumptions [31]. 3D echo by reconstruction of the whole RV overcomes geometrical limitations for the estimation of RV volumes and right ventricular ejection fraction (RVEF). Although RV volumes are slightly underestimated by 3D echo compared with CMR, a good correlation between these two modalities has been reported [94].

## 9. Conclusions

Patients with ARD can have excess CV risk and increased incidence of CV complications. Echocardiography is the first line imaging technique for the detection of cardiovascular involvement and for monitoring the effects of treatment in ARD. Additionally, 3D echo may have an added value in volumetric evaluation especially of the right ventricle, whereas speckle tracking may accurately identify subclinical cardiac dysfunction. It remains to be elucidated in future studies whether the improvement of echo markers of myocardial deformation and microcirculatory function by reduction of the inflammatory burden will reduce adverse cardiac events and improve the prognosis of ARD patients.

## Figures and Tables

**Table 1:** Main echocardiographic findings consistent with cardiovascular involvement in autoimmune rheumatic diseases.

Cardiovascular Manifestations	Abnormalities Consistent with Cardiovascular Involvement	Echocardiographic Parameters for Diagnosis and Assessment of Severity
**Pericarditis**	**Pericardial effusion**	Loculated or circumferential. Mild >10 mm, moderate 10–20 mm, large >20 mm
	**Tamponade**	Early RV diastolic collapse, late RA diastolic collapse, swinging heart, respiratory variation in ventricular chamber size, dilated inferior vena cava. Exaggerated respiratory changes of >25% in mitral inflow and aortic outflow velocity. Respiratory variation of the mitral peak E velocity of >25%
	**Constrictive pericarditis**	Septal bounce, pericardial thickening. Preserved Tissue Doppler e’ velocity >8.0 cm/s
**Myocarditis, ischemic cardiomyopathy**	**Impaired LV systolic function**	Wall motion abnormalities, impaired LVEF
	**LV diastolic dysfunction**	LA volume index >34 mL/m^2^. In patients with normal EF >50%, ratio E/e’ >14, Tissue Doppler e’ velocity of the interventricular septum >7 cm/s or Tissue Doppler e’ velocity of the lateral wall >10 cm/s, TRVmax >2.8 m/s
	**Impaired RV systolic function**	TAPSE >17 mm, FAC >35%, Impaired RVEF (3D echo). S’RV >9.5 cm/s
**Valvular heart disease**	**Valvular abnormalities**	Valve thickening, prolapse of mitral leaflets, valvular nodules in RA, Libman–Sacks vegetations in SLE, Libman–Sacks like vegetations in RA. Moderate or severe valvular regurgitation, rarely stenosis
**Pulmonary hypertension**	**Dilation of right chambers, ventricular interdependence**	RV/LV >1 diameter ratio, flattened interventricular septum, dilated pulmonary artery diameter >25 mm, right atrial area >18 cm^2^, dilated inferior vena cava >21 mm with reduced inspiratory collapse. TRVmax >2.8 m/s and presence of secondary signs suggestive of PH: RV outflow velocity acceleration time >105 m/s, early diastolic pulmonary regurgitation velocity >2.2 m/s
**Aortitis**	**Thickening of aortic walls**	
**Subclinical LV dysfunction Subclinical RV dysfunction**	**Impaired GLS**	GLS >−20%
	**Impaired RV longitudinal strain**	RV free wall Longitudinal Strain >−20%
**Obstructive epicardial CAD and/or coronary microcirculation dysfunction**	**New wall motion abnormalities during stress echo**	
	**Impaired coronary flow reserve**	Coronary Flow Reserve >2

LV: left ventricular, LVEF: left ventricular ejection fraction, LA: left atrial, RA: right atrial, RV: right ventricular, RVEF: right ventricular ejection fraction, 2D: two dimensional, 3D: three dimensional, TAPSE: tricuspid annulus plane systolic excursion, FAC: fractional area change, SLE: systemic lupus erythematosus, RA: rheumatoid arthritis, TRVmax: tricuspid regurgitation maximum velocity, GLS: global longitudinal strain, CAD: coronary artery disease.
